# Atypical Familial Presentation of Chiari 1 Malformation: A Case Report

**DOI:** 10.7759/cureus.82039

**Published:** 2025-04-10

**Authors:** Mandy Hsu, Abdul-Jawad J Majeed, Kyra T Newmaster, Elias Harkins, Gayatra Mainali, Sunil Naik, Sita Paudel

**Affiliations:** 1 Pediatric Neurology, Penn State College of Medicine, Hershey, USA; 2 Neurology, Children's Hospital Los Angeles, Los Angeles, USA; 3 Pediatrics, Penn State Health Children's Hospital, Hershey, USA; 4 Pediatric Neurology, Penn State Health Milton S. Hershey Medical Center, Hershey, USA

**Keywords:** chiari 1 malformation, familial chiari malformation, genetic testing, type 1 chiari malformation, whole genome sequencing

## Abstract

Chari malformations are a group of developmental malformations involving the cerebellum, classified into four subtypes. Type 1 CM (CM1) can cause a heterogenous set of symptoms, ranging from neck pain, occipital headache, and nystagmus to cranial nerve palsies, limb numbness, and cerebellar dysfunction. Diagnosis can be particularly challenging in part due to the lack of understanding surrounding its pathogenesis, though it has been suggested that there may be some genetic component contributing. This report describes a case of familial CM1 in which a mother and her two daughters all demonstrated symptomatic and radiographic evidence of CM1. Notably, the two children presented with symptoms requiring surgical decompression prior to the age of three, which is uncommon. The mother only presented with symptoms after giving birth to her two children. Though the children both had initial symptoms, including difficulty swallowing as well as an established growth hormone deficiency, the mother did not present with such symptoms and did not have any known hypothalamic dysfunction. The pathogenesis of CM1 remains unknown, though it has been suggested that there is some genetic component involved. This report of familial CM1 adds to the body of literature. Familial MRI head screening and whole exome sequencing in individuals with family members showing signs of CM1 could help reveal a genetic pathogenesis for CM1.

## Introduction

Chiari malformations (CMs) are a group of developmental malformations of the cerebellum [1,2]. CMs were first described by John Cleland in 1883 and then further categorized in 1891 into four different subtypes by Hans Chiari [3,4]. Of note, in Type 1 CM (CM1), there is herniation or downward displacement of the cerebellar tonsils into the foramen magnum but without brainstem herniation. It can sometimes result in elevated intracranial pressure. 

The pathogenesis of CM remains a topic of debate, with no one theory capable of explaining all its features. Hans Chiari himself originally proposed a mechanism known now as the hydrodynamic pulsion theory, which assumed that early progressive fetal hydrocephalus caused secondary herniation of the hindbrain. Other existing theories include the molecular genetic theory, crowding theory, and oligo-cerebrospinal fluid theory. CM1, in particular, is thought to be due to either neuroectodermal or mesodermal abnormalities, depending on whether or not it is an isolated disorder. Isolated CM1 is considered to be of mesodermal origin, while CM1 associated with other neurological disorders is thought to be of neuroectodermal origin [5].

Symptomatic CM1 occurs in one out of every 1000 people, but due to an increase in imaging frequency, it is estimated that the prevalence of asymptomatic CM1 is one out of every 100 people [2,6-9]. The phenotypic presentation of CM1 can be vastly heterogeneous. More commonly presenting symptoms include neck pain, occipital headache, cerebellar ataxia, tremors, and nystagmus. If the brainstem is affected, or if there is syringomyelia, cranial nerve palsies and limb numbness or paresthesia may also be presenting symptoms. The initial presentation typically occurs between the ages of 25 and 45 years, with headache as the most common concern [2]. Subsequent imaging confirms the diagnosis, and decompressive surgical treatment is considered if symptoms are debilitating. These concerning symptoms and findings include but are not limited to syringomyelia, myelopathy, occipital cough headache, cranial nerve palsies, and cerebellar dysfunction [10,11].

Diagnosis can be challenging for several reasons. Firstly, the diagnostic criteria are still subject to some debate. Most experts agree that displacement of the cerebellar tonsils below the foramen magnum by 5 mm or more is diagnostic [5]. However, adults can have normal cerebellar tonsils that lie up to 3 mm below the foramen magnum [12]. In the pediatric population, diagnosis is even more challenging, especially for those younger than two years of age, because over 70% of these children present with oropharyngeal dysfunction symptoms, including dysphagia, failure to thrive, reflux, and apnea, rather than headaches or cerebellar symptoms [13-17]. Furthermore, there is no correlation between clinical severity and degree of tonsillar descent, so diagnosis and treatment decisions are typically provider dependent [7,18,19].

While no clear genes have been linked to the pathogenesis of CM1, the condition does sometimes present in families. It is rare, however, for all affected family members to be symptomatic to the extent that they require surgical decompression [20]. In this report, we present a novel case of familial CM1 in a mother and both of her daughters, both of whom required surgical decompression at a young age, thereby adding to the literature supporting a genetic basis for CM1. These children were atypical in that they presented symptomatically under the age of three, highlighting unique features of CM1 presentation in young children.

## Case presentation

Proband

A 15-month-old female patient presented to our institution with several days of progressively unsteady gait, stumbling around, not wanting to walk as much, grabbing at the back of her head, fussiness, unequal pupil size, and somnolence. Her past medical history included tracheomalacia, atrial septal defect, recurrent hypoglycemia secondary to growth hormone deficiency, and failure to thrive. She was born at 36 weeks and six days via an uncomplicated vaginal delivery requiring no neonatal intensive care unit stay. Her motor and cognitive development progressed normally, and she had achieved independent walking and running by 11 months of age. Her family history included a two-year-old sister with CM1 status post-surgical correction and a mother with a cerebellar tonsillar herniation. 

On physical exam, she was alert and active. Pupils were equal, round, and reactive to light with no nystagmus. Cranial nerves II through XII appeared to be intact. Motor exam showed normal muscle tone and bulk in all extremities. Reflexes and coordination were normal. Examination of gait was attempted but then deferred due to the patient either being unwilling or unable to stand and make steps forward.

**Figure 1:**
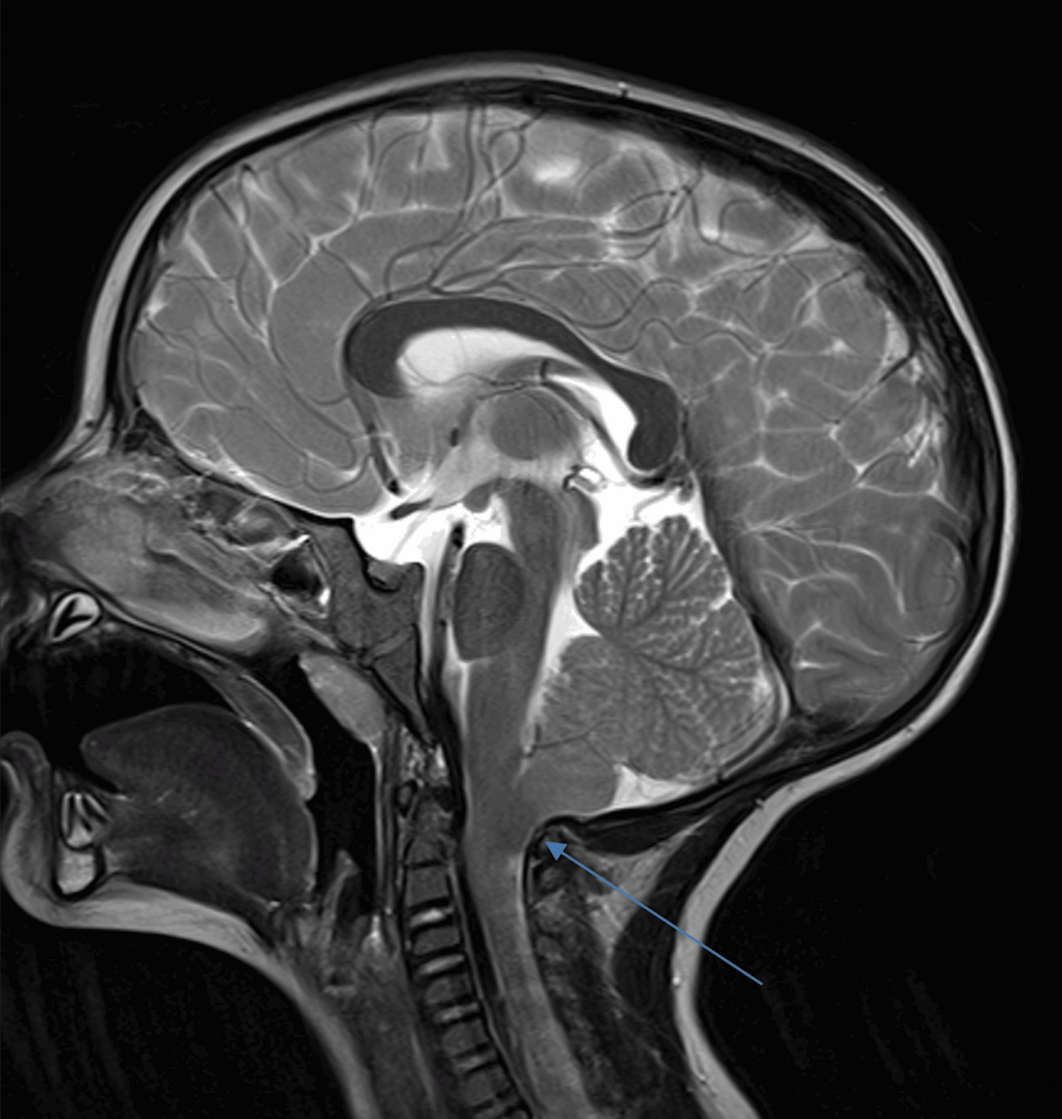
Preoperative sagittal T2 MRI imaging of the proband. Blue arrow indicates herniation of cerebellar tonsils at the foramen magnum.

**Figure 2:**
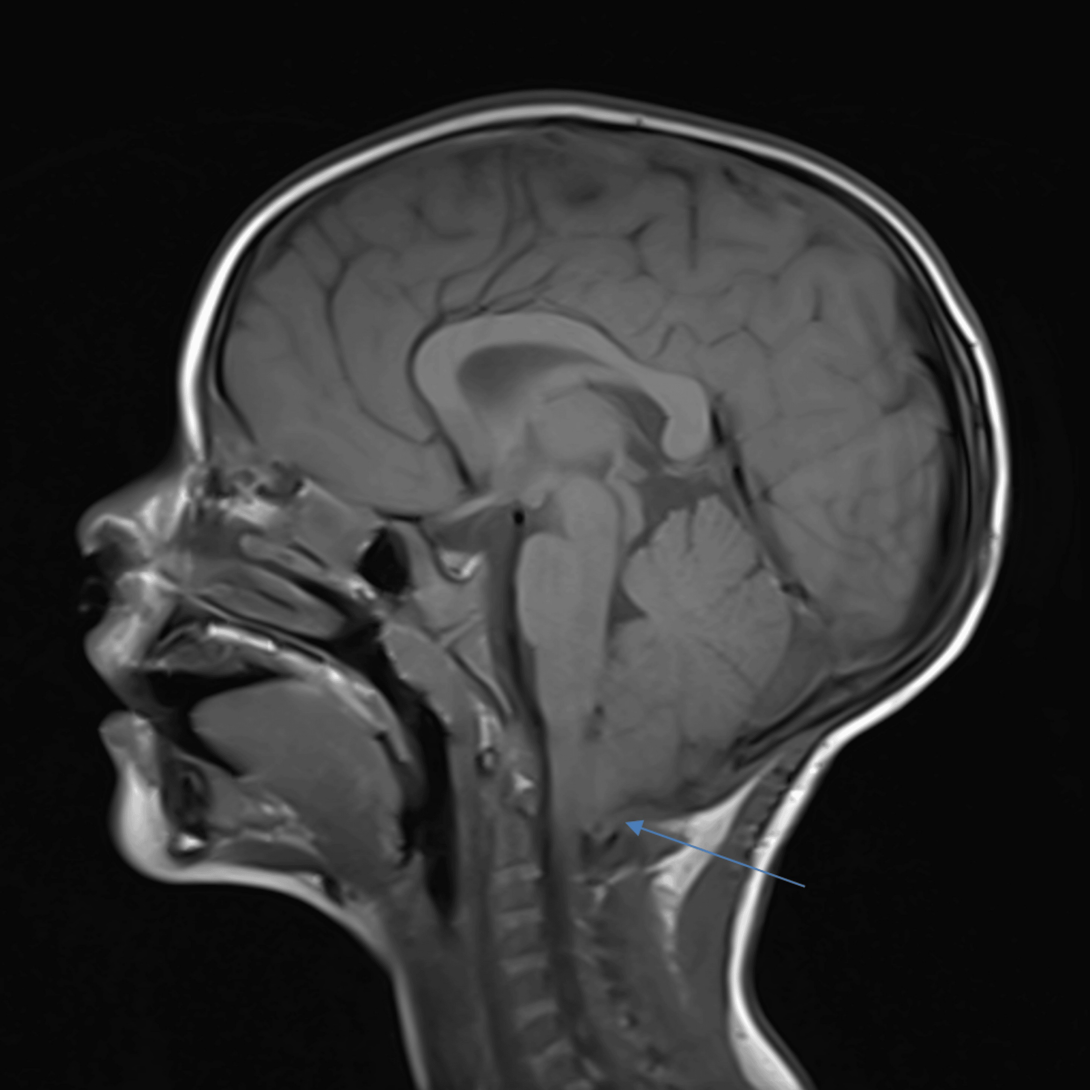
Sagittal T1 MRI imaging of the proband post surgery, demonstrating the expected postoperative changes of a suboccipital craniotomy for Chiari decompression. The blue arrow shows persistent narrowing of the cisterns at the craniocervical junction, unchanged from an earlier MRI two months prior.

Given her gait changes, signs of a possible headache, and family history of CM1, there was high suspicion for CM1 in this patient, so a brain and spine MRI was obtained. MRI showed cerebellar tonsils extending approximately 10 mm below the plane of foramen magnum with diminished cerebrospinal fluid (CSF) flow, confirming a diagnosis of CM1 (Figure 1). She was treated with a suboccipital decompression and C1 laminectomy (Figure 2). Her surgical course was uncomplicated, and she was discharged the following day. Following treatment, though her gait symptoms resolved, she continued to show signs of mild headaches monthly by pointing at her head during times of high exertion. However, the mother noted that she was unsure whether the patient may be copying her sibling about headaches.

Sibling

The course of the proband’s older sister was characterized via chart review, given that familial cases of CM1 are rare. This patient was a two-year-old female child with a past medical history of failure to thrive and recurrent hypoglycemia secondary to growth hormone deficiency at the time of her diagnosis of CM1. She initially presented with shakiness, sweating, pallor, emesis, and a wobbly gait, leading to falls. These episodes had been ongoing for five months prior to her admission. During her hospitalization, she was simultaneously being evaluated for recurrent hypoglycemia by endocrinology and right-sided esotropia by ophthalmology. Though there was high suspicion for a hypoglycemic episode due to her medical history, neurology was consulted for possible seizure-like activity due to her falls. 

Upon physical exam, she was alert and able to speak in short but full sentences. Much of the exam was limited due to irritability and poor participation from the patient. Pupils were equal, round, and reactive to light. Visual fields appeared to be intact. On exam of extra-occular muscles, there appeared to be some possible right cranial nerve VI weakness, but the remainder of cranial nerves II-XII were intact. Motor, strength, reflexes, and coordination were normal, given the limited exam. Overall, the reported nystagmoid movement, ataxia, and upper and lower extremity weakness were not apparent at the time of the exam. 

**Figure 3:**
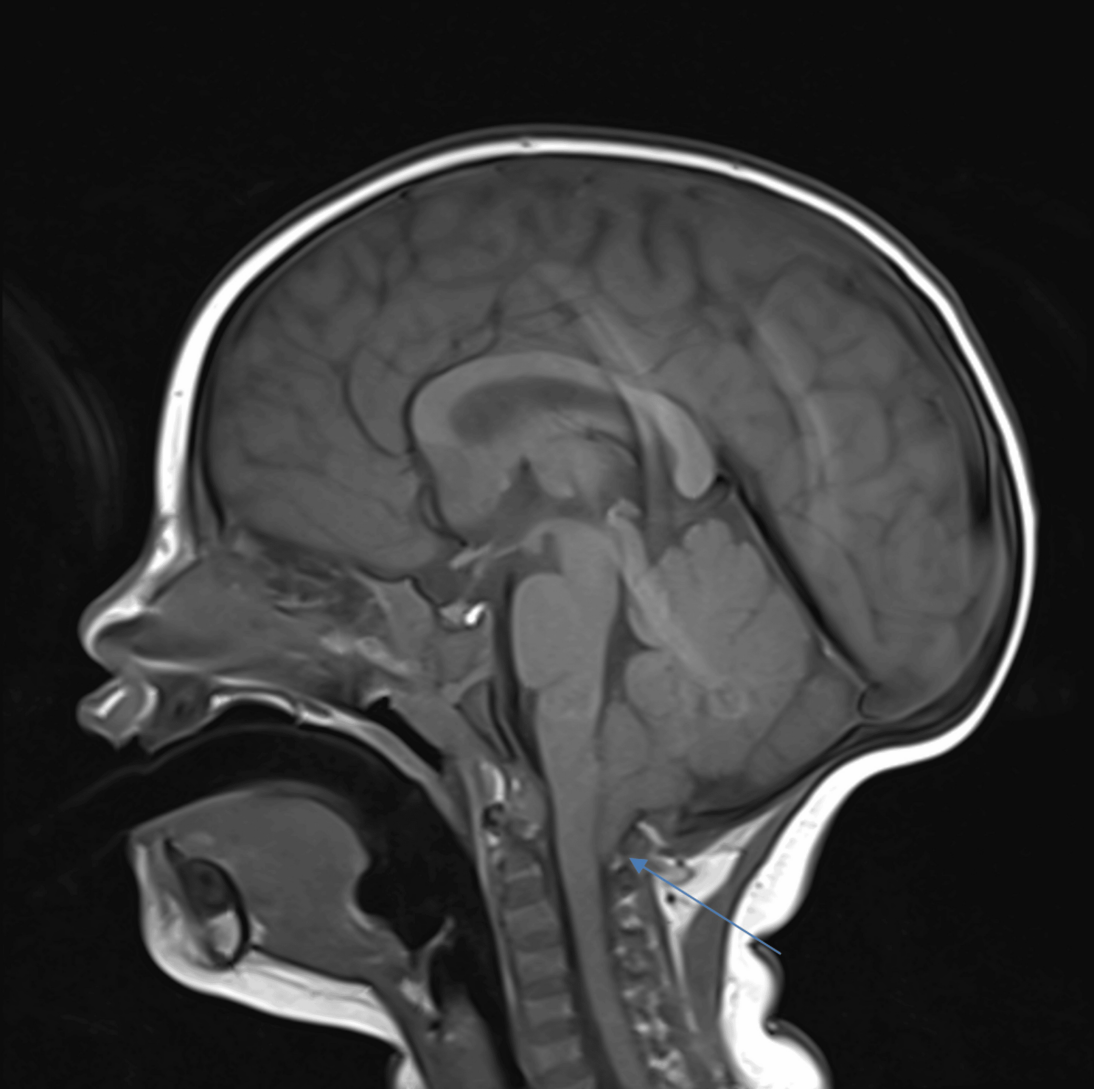
Sagittal T1 MRI imaging of the proband’s sister prior to surgical decompression. The blue arrow indicates cerebellar tonsils extending 10 mm below the foramen magnum.

**Figure 4:**
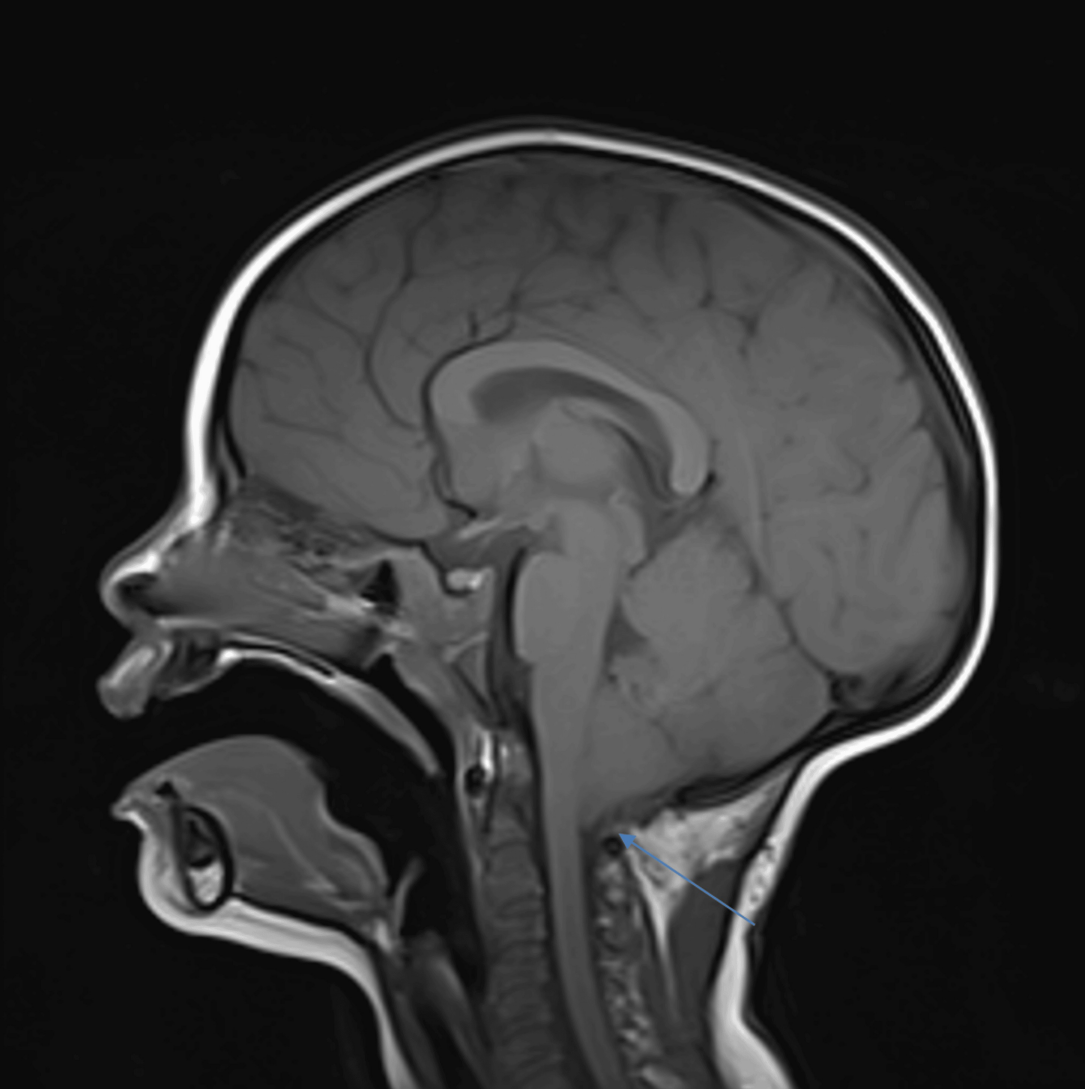
Sagittal T1 MRI imaging of the proband’s sister after surgical decompression, demonstrating improved but still present crowding and cerebellar descent.

An MRI was obtained and demonstrated approximately 8 mm of tonsillar herniation with restricted CSF flow (Figure 3). Afterwards, neurosurgery was consulted, and she was treated with a suboccipital decompression and C1 laminectomy (Figure 4). She had an uncomplicated post-surgical course and was discharged the next day. Since this surgery, she has not demonstrated any ataxia or experienced any other falls; however, she is still receiving treatment for her other medical concerns unrelated to CM1.

Mother

The mother was 23 years old with a past medical history of migraines with vision, speech, and motor symptoms. These symptoms included sensitivity to light and sounds, double vision, tearing from the eyes, bilateral arm weakness and numbness, and slurring of words for a few minutes. She began to experience worsening headaches that were exacerbated by Valsalva maneuvers after giving birth to the proband. Her neurological exam at that time was normal. Her MRI revealed tonsillar herniation 12 mm below the foramen magnum, suggesting CM1; however, it was determined that her headaches were more consistent with migraines and did not necessitate surgical intervention. Most recent surveillance MRI brain with and without contrast show unchanged CM1.

## Discussion

In this case of familial CM1, the mother remained asymptomatic until childbirth, but both her children exhibited initial symptoms such as difficulty swallowing. Uncommonly, both children in this series showed symptoms before the age of three, offering a unique opportunity to explore CM1 symptoms in such a young age group. This report also contributes to the existing literature suggesting an undiscovered genetic etiology for CM1. While growth hormone deficiency has a known correlation with CM1 [21-23], the mother’s lack of hypothalamic dysfunction points to a secondary genetic etiology.

In pediatrics, CM1 can go undiagnosed until late childhood. Although the reason is unclear, it is suggested that infants can have up to 6 mm of herniation in the first decade of life without typical symptoms [12]. Patients under the age of three with CM1 also do not always present with classic symptoms associated with this condition. While headache is a common symptom in pediatric CM1, about one-third of pediatric patients may not develop headaches, and unlike adults, pediatric CM1 headaches are often not occipital or triggered by Valsalva maneuvers or coughing. Contrary to our proband and her sibling, who presented severe symptoms early, typically older children require intervention [7]. Moreover, in younger children or those with developmental delays who have not yet developed full verbal communication, symptomatic CM1 can be especially difficult to diagnose [14,24] and may be mistaken for other conditions. In younger age groups, presentations such as oropharyngeal dysfunction are more common [16], leading to investigations such as swallow studies that do not reveal the malformation. Our proband underwent a similar workup but was diagnosed more quickly by MRI due to her family history of CM1. This emphasizes the importance of family history as a component of the workup in young children presenting with poor feeding or oropharyngeal dysfunction. Though our case report is limited to one family with two very young children with symptomatic presentations of CM1 and an asymptomatic mother, it highlights the importance of considering CM1 in very young children who may not experience or verbalize headaches.

MRI is the gold standard for assessing and diagnosing CM1, and increased usage has led to more identifications of asymptomatic patients [7, 25]. The prognosis of CM1 can be unpredictable, with some patients remaining asymptomatic or even having spontaneous resolution of tonsillar displacement, while others develop worsening syrinx or symptoms requiring surgical intervention [26,27]. Decompressive surgery is currently not indicated unless the patient is symptomatic. If syringomyelia or CSF flow obstruction is present but the patient is asymptomatic, preferred management remains debated. Watchful waiting may be warranted given the unpredictable course of CM1, but in cases of complete CSF flow obstruction, a more aggressive surgical approach may be preferred. Davidson et al. found that over a mean follow-up of about three years, 16.5% of children with incidentally found CM1 required decompressive surgery, and the presence of syringomyelia predicted the eventual need for decompression treatment [26]. It follows that in the context of our familial CM1, head MRI was especially important to consider.

Overall, CM1 is common yet highly heterogeneous, leading to underdiagnosis and hindering the identification of its genetic underpinnings [6-9,25,28,29]. Though CM1 is typically considered to be sporadic, increasing evidence suggests a hereditary component. Our case study adds to this body of evidence. There is limited literature describing familial aggregation of CM1 similar to this presented case [1,3,20,30-32]. These studies show pedigrees with multiple members demonstrating radiological evidence of CM1 with variable symptoms, inheritance patterns, and penetrance. The early symptomatic presentation in our case suggests possible phenotypic anticipation. Imaging of the mother’s parents could support or refute this hypothesis. Some believe that CM1 is unlikely to follow classic Mendelian inheritance patterns and more likely to be polygenic with varying penetrance and include the involvement of non-genetic factors. Other reviews have found associations between CM1 and over 90 Mendelian syndromes [33], highlighting the heterogeneity and uncertainty still surrounding its pathogenesis.

No unique genetic factor has been identified as a cause for isolated CM1. Many associated genes belong to a syndrome, and CM1 heterogeneity complicates genomic investigations. For example, Loukas et al. showed that CM1 is associated with over 50 different genetic syndromes, including connective tissue disorders, disorders of CSF homeostasis, brain overgrowth disorders, and skull development and vascular conditions [34]. Many syndrome-associated genes converge on pathways controlling bone growth, skull patterning, or brain growth [27]. There may also be a unique upstream genetic mechanism, such as one involving chromatin remodeling genes, as shown in a recent exome study by Provenzano in 2021 [29]. Recent whole-genome sequencing on familial CM1 suggests that chromodomain (CHD) gene variants may be involved [35]. Our limited understanding of what appears to be numerous genetic factors involved with CM1 may be shrouding multiple subtypes of the condition.

Despite familial evidence for a genetic etiology, there are no CM1-specific genes on commercially available genetic panels. Given the limited whole-genome sequencing studies on familial CM1 cases, sequencing more patients and families will be necessary to identify if the candidate genes are truly causal [33]. However, the limitations of sequencing should be communicated to patients. In consultation with pediatric genetics, we emphasized to our family the low diagnostic rate of whole-genome sequencing for CM1. They are currently considering whether to pursue this route, given the possibility of negative, uncertain, and unexpected secondary findings.

## Conclusions

This familial case underscores the need for further research into the genetic components of CM1, necessitating large cohort genetic-phenotypic studies. Therefore, if a child is diagnosed with CM1, screening other family members with MRI head imaging and considering genetic testing in families with multiple affected individuals may be beneficial. Finally, this case emphasizes the importance of obtaining a family history of CM1 in children with poor feeding to avoid unnecessary workups, given the numerous presentations of CM1 in pediatric populations.
